# Particle Distribution and Heat Transfer of SiO_2_/Water Nanofluid in the Turbulent Tube Flow

**DOI:** 10.3390/nano12162803

**Published:** 2022-08-15

**Authors:** Ruifang Shi, Jianzhong Lin, Hailin Yang

**Affiliations:** 1 State Key Laboratory of Fluid Power Transmission and Control, Zhejiang University, Hangzhou 310027, China; 2 Faculty of Mechanical Engineering and Mechanics, Ningbo University, Ningbo 315201, China

**Keywords:** SiO_2_/water, pressure drop, particle distribution, heat transfer, turbulent tube flow, numerical simulation

## Abstract

In order to clarify the effect of particle coagulation on the heat transfer properties, the governing equations of nanofluid together with the equation for nanoparticles in the SiO_2_/water nanofluid flowing through a turbulent tube are solved numerically in the range of Reynolds number 3000 ≤ Re ≤ 16,000 and particle volume fraction 0.005 ≤ *φ* ≤ 0.04. Some results are validated by comparing with the experimental results. The effect of particle convection, diffusion, and coagulation on the pressure drop ∆*P*, particle distribution, and heat transfer of nanofluid are analyzed. The main innovation is that it gives the effect of particle coagulation on the pressure drop, particle distribution, and heat transfer. The results showed that ∆*P* increases with the increase in Re and *φ*. When inlet velocity is small, the increase in ∆*P* caused by adding particles is relatively large, and ∆*P* increases most obviously compared with the case of pure water when the inlet velocity is 0.589 m/s and *φ* is 0.004. Particle number concentration *M*_0_ decreases along the flow direction, and *M*_0_ near the wall is decreased to the original 2% and decreased by about 90% in the central area. *M*_0_ increases with increasing Re but with decreasing *φ*, and basically presents a uniform distribution in the core area of the tube. The geometric mean diameter of particle GMD increases with increasing *φ*, but with decreasing Re. GMD is the minimum in the inlet area, and gradually increases along the flow direction. The geometric standard deviation of particle diameter GSD increases sharply at the inlet and decreases in the inlet area, remains almost unchanged in the whole tube, and finally decreases rapidly again at the outlet. The effects of Re and *φ* on the variation in GSD along the flow direction are insignificant. The values of convective heat transfer coefficient *h* and Nusselt number *Nu* are larger for nanofluids than that for pure water. *h* and *Nu* increase with the increase in Re and *φ*. Interestingly, the variation in *φ* from 0.005 to 0.04 has little effect on *h* and *Nu*.

## 1. Introduction

Convective heat transfer plays an important role in industrial and heating/cooling equipment [1]. Enhancing heat transfer to improve the efficiency of heat exchangers is an important research direction. The flow characteristics [2] and geometry of heat transfer surfaces [3,4] are important factors affecting convective heat transfer. Nanoparticles have many important uses [5], for example, it is more effective to add nanoparticles into the fluid (i.e., nanofluid) to improve its thermal conductivity and convective heat transfer coefficient, so as to achieve the purpose of enhancing heat transfer.

The studies on convective heat transfer in nanofluids have been reported in previous research. Ramesh et al. [6] studied the flow and heat transport structures of Al_2_O_3_-Ag and Al_2_O_3_-Cu hybrid nanoparticles in a moving material and observed that the skin friction value of the Al_2_O_3_-Cu/water case was lower in comparison to the values of the Nusselt number for Al_2_O_3_-Ag/water nanoparticles. Ali et al. [7] studied the convective heat transfer of nanofluids with different SiO_2_ volume concentrations in a horizontal tube and found a significant increase in heat transfer with increasing particle concentration. Hejazian et al. [8] used the Euler model and mixture model to study the forced convection of a nanofluid in a tube with a wall exposed to the saturated steam; the results showed that the estimation of convective heat transfer by the hybrid model was in better agreement with the experimental results in the range of Reynolds number 3000 ≤ Re ≤ 14,000 and volume concentration 0.1% ≤ *V*_c_ ≤ 2%.

The effect of the shape and size of the particles on the heat transfer has also been investigated. Liu et al. [9] introduced two new shape parameters, flatness and elongation, to study the effect of non-spherical particles on the heat transfer and established a new modified model for the correlation of Nusselt numbers related to particle shape, size, and volume fraction. Hemmat and Saedodin [10] conducted the empirical study on heat transfer and thermophysical properties considering different diameters of MgO nanoparticles, and the results showed that the traditional formulation underestimated the thermophysical parameters such as thermal conductivity and dynamic viscosity. They also found that the smaller the diameter of the nanoparticle, the higher the thermal conductivity and kinetic viscosity, and the larger the convective heat transfer coefficient. Keblinski et al. [11] gave four explanations for the increase in the thermal conductivity with decreasing nanoparticle scale, namely, the Brownian motion of the nanoparticles, the molecular layer of the liquid at the liquid and particle interfaces, the nature of heat transfer in the nanoparticles, and particle aggregation. Bahiraei also [12] demonstrated the effect of the thermophoretic force and the Brownian force on the convection heat exchange through numerical simulation and found that its effect was more obvious far from the pipe inlet. Heyhat and Kowsary [13] used the Buongiorno model to study the flow of Al_2_O_3_-water nanofluid through a pipeline, and found that compared with the uniform volume fraction, the heat transfer coefficient increased; thus, realizing the same enhanced convection heat transfer.

Lin et al. [14,15] analyzed the characteristic of heat transfer in the flow of nanofluids with spherical and rod-like nanoparticles and found that it was more effective to enhance the heat transfer for the rod-like nanoparticles with larger aspect ratio at the flow with higher Reynolds number. Zhang [16] studied the heat transfer in a turbulent curved tube and found that the energy performance evaluation standard ratio of Al_2_O_3_/water nanofluid to base fluid increased with the increase in Reynolds number, volume fraction, and Dean number, but with the decrease in Schmidt number. Calvino et al. [17] analyzed experimentally the convective heat transfer performance of a polycarboxylate chemically modified GnP dispersion in water at 0.50 wt% and obtained the convective heat transfer coefficients reaching enhancements for the nanofluid of up to 13%. Alam et al. [18] presented the experimental forced convective heat transfer coefficient of nanorods zinc oxide–ethylene glycol nanofluids in laminar flow. The results showed that adding only small amounts of zinc oxide nanorods to ethylene glycol could significantly increase the heat transfer coefficient.

At present, there are some models and solutions for nanofluids. Jamshed et al. [19] chose the Prandtl–Eyring hybrid nanofluid as a working fluid in the solar water pump model for the production of a solar water pump in a parabolic trough surface collector. Shahzad et al. [20] developed models for coronavirus disease at different stages with the addition of more parameters due to interactions among the individuals and computed the local sensitivities for each model state concerning the model parameters using the model reduction techniques. Abdullah et al. [21] developed a new mathematical model by including the resistive class together with the quarantine class and used it to investigate the transmission dynamics of the novel coronavirus disease.

By reviewing previous relevant research, it can be seen that the there are few studies on the nanoparticle coagulation in the nanofluids and resulting distribution of particles, and the research on the effect of particle coagulation on the heat transfer characteristics is lacking. However, nanoparticle coagulation is a common phenomenon in nanofluid flow. After nanoparticle coagulation, the number and size of particles in the flow will be changed, which will affect the pressure drop and heat transfer. Therefore, in the present work, we study numerically the momentum and energy equations of nanofluids together with the population balance equation for nanoparticles in the SiO_2_/water nanofluid flowing through a turbulent tube and explore the effect of particle coagulation on the pressure drop, particle distribution, and heat transfer of SiO_2_/water nanofluids.

## 2. Basic Equations

**Figure 1:**
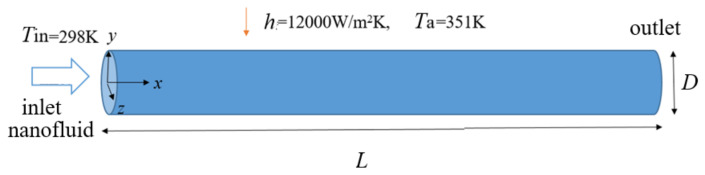
The geometry of the tube and coordinate system.

The turbulent tube flow of nanofluids with SiO_2_ nanoparticles is simulated numerically with saturated steam wall exposure (convective boundary conditions). We chose SiO_2_ as the nanoparticles because it is insoluble in water and has good dispersion and stability in water. Figure 1 shows the geometry of the tube with a diameter *D* = 0.0064 m and length *L* = 1.5 m. The tube is made of copper material and has a wall thickness of 0.0008 m.

### 2.1. Equations for the Nanofluid

The nanofluid is the mixture of the base liquid with the nanoparticles. The governing equations for the nanofluid are expressed as [22,23]:

(1) $$\frac{\partial \left(\rho_{nf}u_{i}\right)}{\partial x_{i}}=0$$

(2) $$\frac{\partial }{\partial t}\left(\rho_{nf}u_{i}\right)+\frac{\partial }{\partial x_{j}}\left(\rho_{nf}u_{i}u_{j}\right)=-\frac{\partial p}{\partial x_{i}}+\frac{\partial }{\partial x_{j}}\left(\mu_{nf}\left(\frac{\partial u_{i}}{\partial x_{j}}+\frac{\partial u_{j}}{\partial x_{i}}\right)\right)+\frac{\partial }{\partial x_{j}}\left(-\rho_{nf}\bar{u_{i}^{\prime }u_{j}^{\prime }}\right)$$

(3) $$\frac{\partial }{\partial t}(\rho_{nf}E)+\frac{\partial }{\partial x_{i}}\left[u_{i}(\rho_{nf}E+p)\right]=\frac{\partial }{\partial x_{j}}\left[\left(k_{nf}+\frac{c_{p,nf}\mu_{t}}{{\mathrm{Pr}}_{t}}\right)\frac{\partial T}{\partial x_{j}}\right]$$

where *E* is total energy $E=c_{p,nf}T-(P/\rho_{nf})+\left(u^{2}/2\right)$; *T* is nanofluid temperature;$\rho_{nf},\mu_{nf},c_{p,nf},k_{nf}$ are density, dynamic viscosity, specific heat capacity, and thermal conductivity of the nanofluid, respectively; *u_i_* is nanofluid velocity. The last term on the right-hand side of Equation (2) is the Reynolds stress *R_ij_* [15]:

(4) $$R_{ij}=-\rho_{nf}\bar{u_{i}{}^{\prime }u_{j}{}^{\prime }}=\mu_{t}\left(\frac{\partial u_{i}}{\partial x_{j}}+\frac{\partial u_{j}}{\partial x_{i}}\right)-\frac{2}{3}\rho_{nf}k\delta_{ij}-\frac{2}{3}\mu_{t}\frac{\partial u_{k}}{\partial x_{k}}\delta_{ij}$$

where *μ_t_* is turbulent dynamical viscosity and *δ* is the Kronecker function.

The equations for the turbulent dissipation rate ω and turbulent kinetic energy *k* are given by [3,24]:

(5) $$\frac{d}{dt}(\rho \omega )=\nabla \cdot \left(\rho D_{\omega }\nabla \omega \right)+\frac{\rho \gamma G}{\nu_{t}}-\frac{2}{3}\rho \gamma \omega (\nabla \cdot u)-\rho \beta \omega^{2}-\rho \left(F_{1}-1\right)CD_{k\omega }+S_{\omega }$$

(6) $$\frac{d}{dt}(\rho k)=\nabla \cdot \left(\rho D_{k}\nabla k\right)+\rho G-\frac{2}{3}\rho k(\nabla \cdot u)-\rho \beta^{*}\omega k+S_{k}$$

where *G* is turbulent kinetic energy generation rate due to the anisotropic part of the Reynolds stress tensor; *D_k_*, *D_ω_* represent the effective diffusion of *k* and *ω*; *S_k_*, *S_ω_* are the internal source terms of *k* and *ω*; the turbulent kinematic viscosity is obtained by:

(7) $$\nu_{t}=a_{1}\frac{k}{\max\left(a_{1}\omega ,b_{1}F_{2}S\right)}$$

where **S** is a measure of the strain rate tensor, the other default model constants are listed in Table 1, and the auxiliary relationships are as follows:

$$\begin{array}{l} F_{1}=\tanh\left\{{\left\{\min\left[\max\left(\frac{\sqrt{k}}{\beta^{*}\omega y},\frac{500v}{y^{2}\omega }\right),\frac{4\rho \sigma_{\omega 2}k}{CD_{k\omega }y^{2}}\right]\right\}}^{4}\right\},CD_{kw}=\max\left(2\rho \sigma_{\omega 2}\frac{1}{\omega }\frac{\partial k}{\partial x_{i}}\frac{\partial \omega }{\partial x_{i}},10^{-10}\right)\\ F_{2}=\tanh\left\{{\left[\max\left(\frac{2\sqrt{k}}{\beta^{*}\omega y},\frac{500v}{y^{2}\omega }\right)\right]}^{2}\right\},G=-\bar{\rho u_{i}^{\prime }u_{j}^{\prime }}\left(\partial u_{j}/\partial x_{i}\right),\gamma =\gamma_{1}F_{1}+\gamma_{2}\left(1-F_{1}\right)\\ D_{k}=\mu +\frac{\mu_{t}}{\alpha_{k}},D_{\omega }=\mu +\frac{\mu_{t}}{\alpha_{\omega }}\\ \alpha_{k}=\frac{1}{F_{1}/\alpha_{k1}+\left(1-F_{1}\right)/\alpha_{k2}},\alpha_{\omega }=\frac{1}{F_{1}/\alpha_{\omega 1}+\left(1-F_{1}\right)/\alpha_{\omega 2}} \end{array}$$

### 2.2. Population Balance Equation for Nanoparticles

The population balance equation essentially explains the various ways in which state-specific particles form or disappear from the system [25]. The population balance equation obtained by considering the evolution of nanoparticles under convection, diffusion, and coagulation is:

(8) $$\begin{array}{ll} \frac{\partial n\left(v,t\right)}{\partial t}+u\cdot \nabla n\left(v,t\right)-\nabla \cdot \left(D_{B}+\nu_{t}\right)\nabla n\left(v,t\right) & =\frac{1}{2}\int_{0}^{v}\beta (v_{1},v-v_{1})n\left(v_{1},t\right)n\left(v-v_{1},t\right)dv_{1}\\ & -\int_{0}^{\infty }\beta (v_{1},v)n\left(v_{1},t\right)n\left(v,t\right)dv_{1} \end{array}$$

where n(v,t) is particle size distribution function, reflecting the spatial distribution of the number of particles with a volume of *v* at time *t*; the diffusion coefficient caused by the particle Brownian motion is expressed as $D_{B}=k_{B}T/3\pi \mu_{nf}d_{p}$; $\beta (v_{1},v)$ is coagulation kernel function of two particles with volumes of v and $v_{1}$ under the action of collision, and the coagulation of nanoparticles mainly results from particle Brownian motion.

The particle distribution in the above equations should be solved for the systems with different particle types, so the computational cost is too high. For this reason, the moment method, the partition method, and the Monte Carlo method are developed, among which the moment method is most commonly used, but it needs to solve the problem of equation closure. The integral moment method for the equilibrium equation of particles is collated by Li et al. [26]. According to the concept of the moment method proposed by Hulbert and Katz [27], the moment is defined as $m_{k}=\int_{0}^{\infty }n\left(v,t\right)v^{k}dv$, and the *k*-order moment equation of the particle size distribution obtained by integrating Equation (8) is:

(9) $$\frac{\partial m_{k}}{\partial t}+u_{i}\frac{\partial m_{k}}{\partial x_{i}}-\frac{\partial }{\partial x_{i}}\left[\left(D_{B}+\nu_{t}\right)\frac{\partial m_{k}}{\partial x_{i}}\right]=\frac{1}{2}\int_{0}^{\infty }\int_{0}^{\infty }\left[{\left(v+v_{1}\right)}^{k}-v^{k}-v_{1}^{k}\right]\beta (v_{1},v)n(v,t)n(v_{1},t)dvdv_{1}$$

The distribution of particles in the gas medium can be divided into free molecular zones, transition zones, sliding zones, and continuous zones according to different Knudsen numbers (the ratio of the mean free path to the particle diameter) [28]. However, the mean free path of liquid molecules in solid–liquid systems is the same as the radius of 0.1 nm particle scale, and nanoparticles in a liquid can usually be modeled with standard continuum theory. The expression of the corresponding particle coagulation kernel function is:

(10) $$\beta (v_{1},v)=\frac{2k_{B}T}{3\mu_{nf}}\left(\frac{1}{v^{1/3}}+\frac{1}{v_{1}^{1/3}}\right)\left(v^{1/3}+v_{1}^{1/3}\right)$$

By substituting Equation (10) into Equation (9) and closing the moment equation with Taylor’s series approximation technique [29] which is an effective method [30,31], we obtain the first three closed moment equations as follows:

(11) $$\frac{\partial m_{0}}{\partial t}+u_{i}\frac{\partial m_{0}}{\partial x_{i}}-\frac{\partial }{\partial x_{i}}\left[\left(D_{B}+\nu_{t}\right)\frac{\partial m_{0}}{\partial x_{i}}\right]=\frac{2k_{B}T}{3\mu_{nf}}\left[\frac{2m_{0}^{4}m_{2}^{2}-13m_{0}^{3}m_{1}^{2}m_{2}-151m_{0}^{2}m_{1}^{4}}{81m_{1}^{4}}\right]$$

(12) $$\frac{\partial m_{1}}{\partial t}+u_{i}\frac{\partial m_{1}}{\partial x_{i}}-\frac{\partial }{\partial x_{i}}\left[\left(D_{B}+\nu_{t}\right)\frac{\partial m_{1}}{\partial x_{i}}\right]=0$$

(13) $$\frac{\partial m_{2}}{\partial t}+u_{i}\frac{\partial m_{2}}{\partial x_{i}}-\frac{\partial }{\partial x_{i}}\left[\left(D_{B}+\nu_{t}\right)\frac{\partial m_{2}}{\partial x_{i}}\right]=\frac{4k_{B}T}{3\mu_{nf}}\left[\frac{2m_{0}^{4}m_{2}^{2}-13m_{0}m_{1}^{2}m_{2}-151m_{1}^{4}}{81m_{1}^{2}}\right]$$

### 2.3. Thermophysical Parameters of the Nanofluid

In this study, the properties of the basic fluid and the nanoparticles are assumed to be unchanged with temperature, and the thermophysical parameters of the material are shown in Table 2.

The effective density and specific heat capacity of nanofluid are given by the concept of hybrid theory commonly used [32,33]:

(14) $$\rho_{nf}=\varphi \rho_{p}+(1-\varphi )\rho_{f}$$

(15) $$c_{p,nf}=\frac{\varphi c_{p,p}\rho_{p}+(1-\varphi )\rho_{f}c_{p,f}}{\rho_{nf}}\;$$

where $\rho_{f},\rho_{p}$ are densities of the base fluid and nanoparticles at temperature 298 K, respectively; $c_{p,f},c_{p,p}$ are the specific heat capacities of the base fluid and nanoparticles, respectively; *φ* is the volume fraction of the nanoparticles.

The properties of heat transfer in the nanofluid are improved by reducing the heat resistance when adding nanoparticles of high thermal conductivity to the basic fluid. The calculation of the thermal conductivity and dynamic viscosity has developed different expressions in terms of theoretical and empirical corrections [34,35,36]. The effective thermal conductivity of the classical Maxwell theory model is calculated by:

(16) $$k_{nf}=\frac{k_{p}+2k_{f}+2\varphi \left(k_{p}-k_{f}\right)}{k_{p}+2k_{f}-\varphi \left(k_{p}-k_{f}\right)}k_{f}$$

where $k_{f},k_{p}$ are thermal conductivity of the base fluid and nanoparticles, respectively.

Experimental results of nanofluid viscosity incorporating particles of different sizes, shapes, and volume fractions showed that the Einstein equation is significantly underestimated by the increased viscosity as commonly used in the literature [37]. Therefore, the experimental modified model proposed by Corcione [38] with relatively small error is used to predict effective viscosity:

(17) $$\mu_{nf}=\frac{\mu_{f}}{1-34.87{\left(d_{p}/d_{f}\right)}^{-0.3}\varphi^{1.03}}$$

where *μ_f_* is dynamic viscosity of the base fluid; *d_p_* is nanoparticle diameter; *d_f_* is equivalent diameter of the base fluid molecule given by $d_{f}={\left(6M/N_{A}\pi \rho_{f}\right)}^{1/3}$, where *M* is molecular weight of the base fluid (18 g/mol) and *N*_A_ is Avogadro constant (6.022140857 × 10^23^ mol^−1^).

Reynolds number and Prandtl number of nanofluids are Re=ρnfuinD/μnf and $\mathrm{Pr}=\mu_{nf}C_{p,nf}/k_{nf}$. Table 3 summarizes the parameters of nanofluids at different volume fractions.

## 3. Numerical Method and Verification

### 3.1. Numerical Method

To solve the above-mentioned system of control equations, a solver is customized on the OpenFoam platform based on the finite volume method. The SIMPLE algorithm is used to solve the coupling problem of velocity and pressure, and to synchronize the velocity field and temperature field solved in the flow to the moment Equation (11–13) to obtain the information of the particle field. The time term in the equation is discretized in the Euler scheme, the gradient term is a Gaussian linear dispersion, the vector convection term is treated in the Gauss linear upwind limited scheme, and the scalar transport convection term is treated in the Gauss limited linear way.

In the computation, the O-type hexahedron structured mesh division method is adopted for the tube. The grid nodes on the square edge of the section core are evenly distributed, the square vertex to the circular wall grid node *b* is unevenly distributed, the grid near the wall is tighter, and the axis-up grid node *c* is evenly distributed.

### 3.2. Boundary Condition

The boundary conditions used to solve the equations are as follows:

1. Inlet

The boundary condition for velocity is selected, the inlet temperature is set to be 298K, and the volume fraction of the particles is uniform.

2. Wall

No-slip boundary condition is used for velocity. For the outside of the wall and the sub-saturated steam contact, the ambient temperature *T*_steam_ = 351 K, and the heat transfer coefficient for steam *h*_steam_ = 12,000 W/m^2^K. The volume fraction of the particles is zero gradient

3. Outlet

The gradient of each parameter at the outlet is zero.

### 3.3. Parameter Definition

The pressure drop ∆*P* from inlet to outlet is defined as:

(18) $$\Delta P=P_{\mathrm{in}}-P_{\mathrm{out}}$$

The convective heat transfer coefficient *h* and Nusselt number *Nu* are defined as:

(19) $$h=\frac{{\left(\rho C_{p}\right)}_{\mathrm{nf}}\cdot A\cdot u\left(T_{\mathrm{out}}-T_{\mathrm{in}}\right)}{\pi \cdot D\cdot L\left(T_{w}-T_{b}\right)},\;\mathrm{Nu}=\frac{h\cdot D}{k_{\mathrm{nf}}}$$

where *A* is cross-sectional area of the tube; $T_{w},T_{in},T_{out},T_{b}$ are mean wall temperature, mean inlet temperature, mean outlet temperature, and volume temperature, respectively, $T_{b}=\left(T_{\mathrm{in}}+T_{\mathrm{out}}\right)/2$.

### 3.4. Main Steps of the Numerical Simulation

(1) Solve Equations (1)–(7) with φ= 0 to get *u_i_*.
(2) Solve Equations (11)–(13) to get *m*_0_, *m*_1_, and φ.
(3) Substitute φ into Equations (14)–(17) to get *ρ_nf_*, *c_p,nf_*, *k_nf_*, and *μ_nf_*.
(4) Substitute φ, *ρ_nf_*, *c_p,nf_*, *k_nf_*, and *μ_nf_* into Equations (1)–(7), and solve the equations to get *u_i_*, *p*, and *T*.
(5) Repeat steps (2) to (4) based on the new flow velocity *u_i_* until the difference between the successive results of *u_i_*, *p*, and *T* is less than a definite value.
(6) Calculate the pressure drop ∆*P* based on Equation (18), *h* and Nu based on Equation (19).

### 3.5. Grid Independence and Verification of Calculation Methods

The grid information and grid independence are shown in Table 4 where the pressure drop ∆*P* and convective heat transfer coefficient *h* are shown. The comparison of ∆*P* and *h*_mean_ shows that the deviation of ∆*P* and *h*_mean_ is small for 20 × 15 × 1500 (Grid 2) and 24 × 18 × 1500 (Grid 3), the deviations of *h*_mean_ and ∆*P* are within 0.06% and 0.6%, respectively. For 20 × 15 × 1500 (Grid 2) and 20 × 15 × 150 (Grid 4), the deviations of *h*_mean_ and ∆*P* are within 0.5% and 0.2%, respectively. In order to balance the calculation time and accuracy, Grid 4 is selected for the following computation.

**Figure 2:**
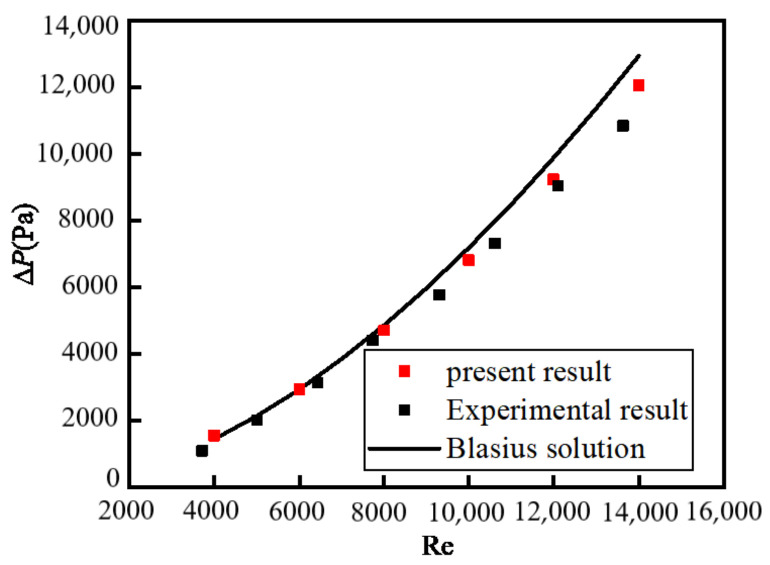
Relationship between *∆P* and Re.

**Figure 3:**
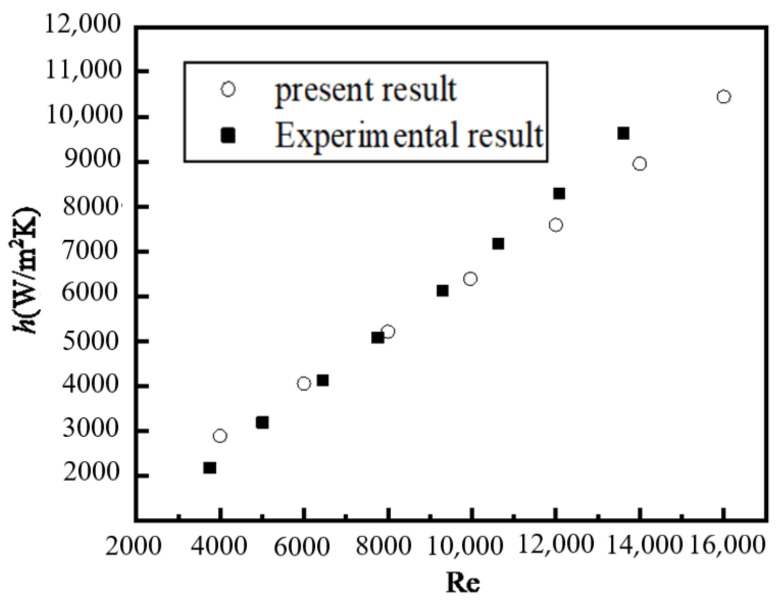
Relationship between *h* and Re.

The variations in ∆*P* and mean heat transfer coefficient *h*_mean_ with Reynolds number for pure water in the tube flow are shown in Figure 2 and Figure 3 where the present numerical results, experimental results [37], and Blasius solution (as seen in Equation (20)) are given as a comparison. We can see that the three results agree well.

(20) $$\Delta P=fL\rho_{nf}u_{in}^{2}/2D=0.158L\rho_{nf}^{0.75}\mu_{nf}^{0.25}D^{-1.25}u_{in}^{1.75}$$

## 4. Results and Discussion

The equations are solved numerically in the range of Reynolds number 3000 ≤ Re ≤ 16,000 and particle volume fraction 0.005 ≤ *φ* ≤ 0.04. The selection of the range of Re and *φ* is based on the most common operating conditions in practical applications.

### 4.1. Pressure Drop

During the process of nanofluid flowing through the tube, energy loss will occur due to the action of viscous force. The empirical formula of the frictional coefficient is used to measure the energy loss. Nikuradse’s experiments on the flow with different tube diameters and flux revealed the law of flow energy loss in the tube and gave the relationship between the energy loss coefficient and the relative roughness as well as Reynolds number. Five regions were divided according to the Nikuradse’s experimental result, the region calculated in this paper belongs to the turbulent smooth region in which the energy loss coefficient along the tube is independent of the relative roughness, only related to the Reynolds number, and given by the Blasius formula f=0.3164Re−0.25.

**Figure 4:**
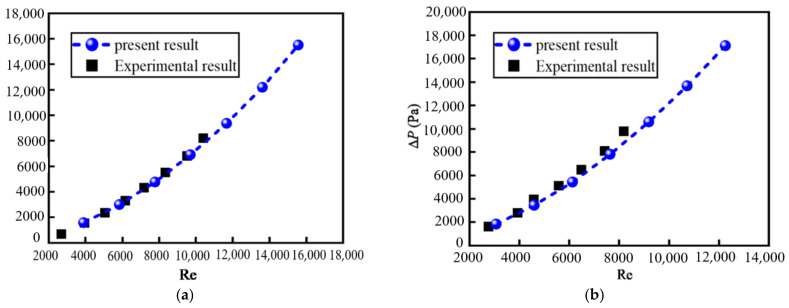
Relationship between ∆*P* and Re with different *φ:* (**a**) *φ* = 0.005; (**b**) *φ* = 0.04.

Figure 4 shows the relationship between the pressure drop ∆*P* and Reynolds number Re with different volume fractions *φ* of nanoparticles. The present numerical results are in good agreement with the experimental results [37]. The pressure decreases along the flow direction due to the energy loss when nanofluid flows through the tube. This energy loss is caused by overcoming the internal friction and the collision and momentum exchange between the fluid particles in the turbulent flow. The values of ∆*P* increase with the increase in Re. The higher the Re is, the more intense the collision and momentum exchange between the fluid particles is, the greater the energy loss is, so the larger ∆*P* is.

**Figure 5:**
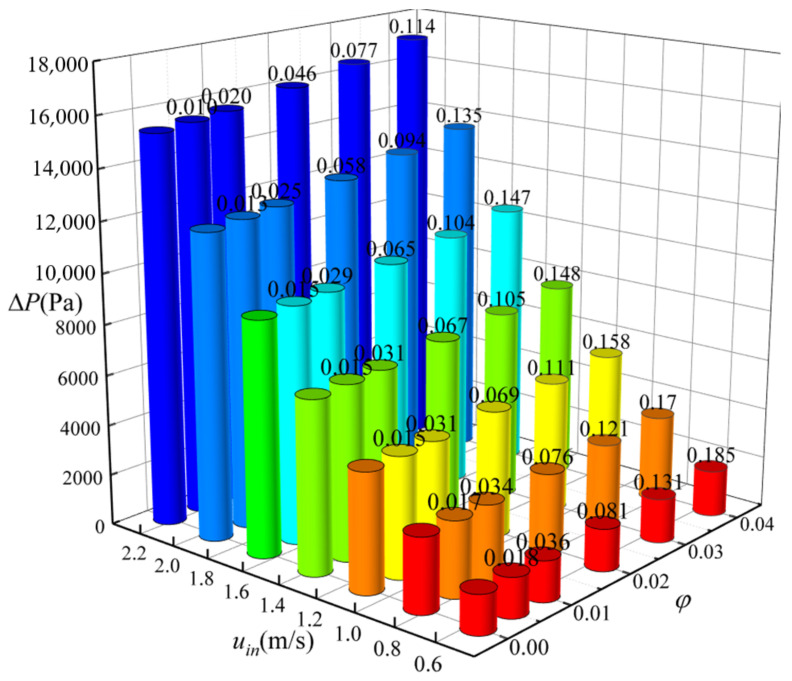
Relationship between ∆*P* and fluid velocity as well as *φ*.

It can be seen from Figure 5 that, at the same inlet velocity, the values of ∆*P* increase significantly after adding nanoparticles because particles added in the base fluid would increase the flow resistance. ∆*P* is directly proportional to *φ*, which is attributed to the following two aspects: 1) the viscosity of the nanofluid increases with the increase in *φ*, which hinders the motion of the nanofluid; 2) irregular motion and migration of particles are enhanced with increasing *φ*. For a specific *φ*, the larger the inlet velocity is, the larger the value of ∆*P* is. In Equation (20), ∆*P* is proportional to the 1.75 power of the velocity in the turbulent smooth region. On the other hand, the larger the inlet velocity, the more intense the friction of the fluid with the tube wall, so the more energy is lost. When the inlet velocity is small, the increase in ∆*P* caused by adding particles is relatively large. The value of ∆*P* increases most obviously compared with the case of pure water when the inlet velocity is 0.589 m/s and *φ* is 0.004.

### 4.2. Particle Distribution

In addition to the influence of *φ* on the viscosity and heat transfer of nanofluids, the influence of particle size and shape is also considered. This means that we need to consider whether nanoparticles coagulate and thus change the particle size. Prasher [39] combined the coagulate kinetics of colloidal solutions at nanometer scale with heat transfer physics and found that the particle coagulation can explain the various abnormal phenomena observed in the experiment well, for example, the thermal conductivity of nanofluids has a peak value, and the dependence of thermal conductivity on temperature, time, and pH value increases with the increase in relative particle size.

Here, we analyze the particle distribution at different *φ* considering particle coagulation. At the inlet, the nanoparticles are single-mode monodisperse (monomers of the same size, and the initial particle diameter is the same), and the the particle diameters meet the logarithmic normal distribution at the outlet after particle coagulation [40]. The geometric mean diameter of the particle (GMD) and the geometric standard deviation of the diameter (GSD) are expressed as:

(21) $$\mathrm{GMD}={\left(M_{1}{}^{2}/\sqrt{M_{0}{}^{3}M_{2}}\right)}^{1/3}$$

(22) $$\mathrm{GSD}=\exp\left(\sqrt{\ln\left(M_{0}M_{2}/M_{1}{}^{2}\right)}/3\right)$$

where $M_{0},M_{1},M_{2}$ are dimensionless particle number concentration, volume concentration, and the second moment of particles, respectively:

(23) $$M_{0}=m_{0}/m_{00},\;M_{1}=m_{1}/m_{10},\;M_{2}=m_{2}/m_{20}$$

where *m*_0_ and *m*_00_ are the particle number concentration and initial particle number concentration; *m*_1_ and *m*_10_ are the volume concentration and initial volume concentration; *m*_2_ and *m*_20_ are the second moment and initial second moment, respectively.

#### 4.2.1. Distribution of Particle Number Concentration

**Figure 6:**
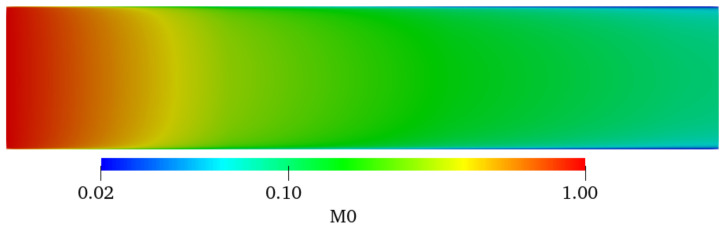
Distribution of *M*_0_ in the region of 0 ≤ x ≤ 5*D* (Re = 8819, *φ* = 0.02).

The distribution of particle number concentration is determined using particle convection, diffusion, and coagulation. Particle convection and diffusion do not change the total particle number, but particle coagulation reduces the total particle number. The characteristic time of coagulation is very short. As shown in Equation (23), the zero-order moment *M*_0_ represents the particle number concentration. The distribution of *M*_0_ in the region of 0 ≤ *x* ≤ 5*D* is shown in Figure 6 where a significant reduction in *M*_0_ was observed because of particle coagulation. The value of *M*_0_ near the wall of the tube decreased to the original 2%, and it also decreased by about 90% in the central area of the tube.

**Figure 7:**
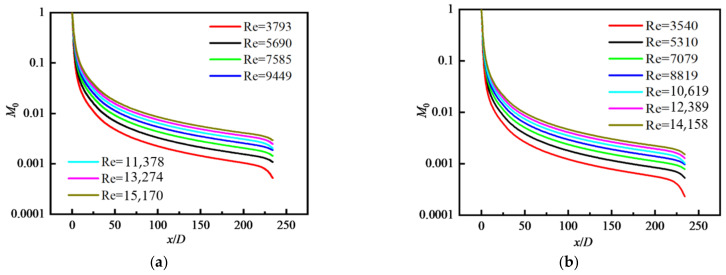

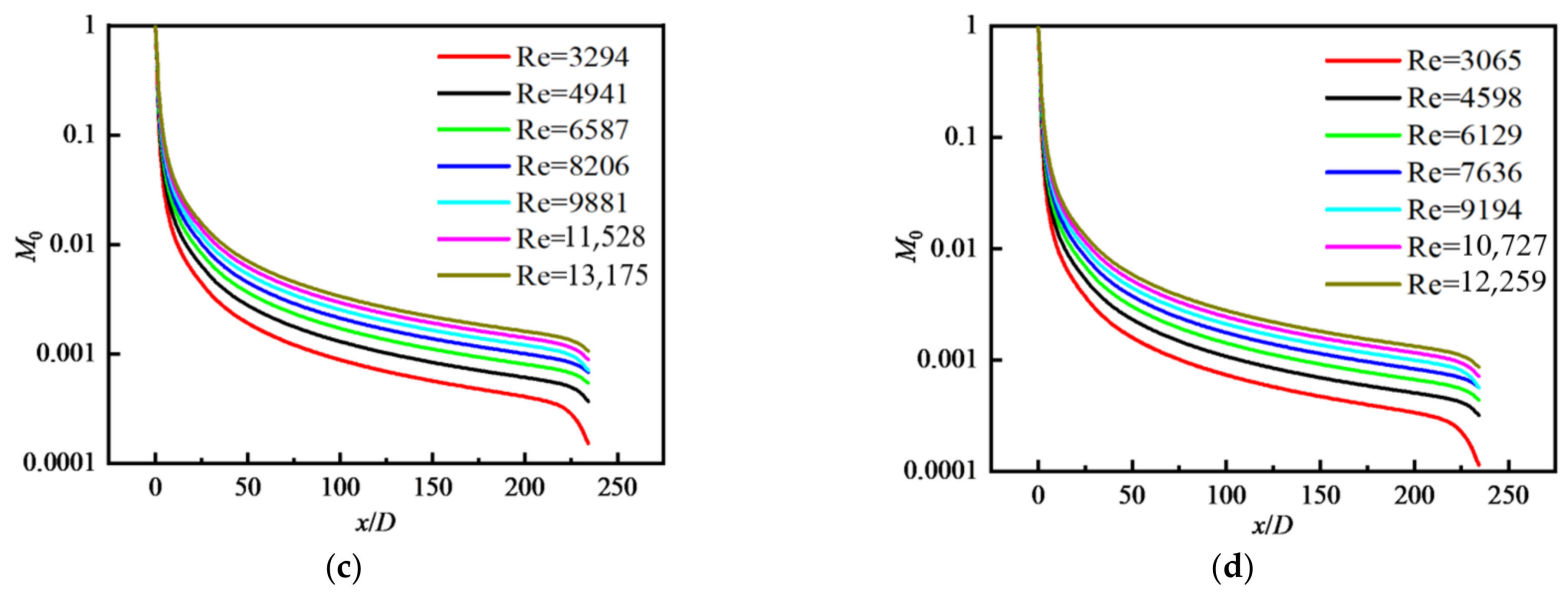
Distribution of *M*_0_ along the flow direction with different *φ*: (**a**) *φ* = 0.01; (**b**) *φ* = 0.02; (**c**) *φ* = 0.03; (**d**) *φ* = 0.04.

Figure 7 shows the distribution of *M*_0_ along the flow direction under different Re and *φ*, respectively. *M*_0_ is averaged over the cross-section. It can be seen that *M*_0_ decreases along the flow direction, and the reduction rate gradually slows down. For a fixed *φ*, with the increase in Re, the values of *M*_0_ increase and the reduction rate of *M*_0_ along the flow direction decreases. Comparing the case with different *φ*, we can see that *M*_0_ increases with the increase in Re, while *M*_0_ decreases with the increase in *φ* because the larger the value of *φ*, the greater the probability of particle collision and coagulation, so the smaller the value of *M*_0_.

**Figure 8:**
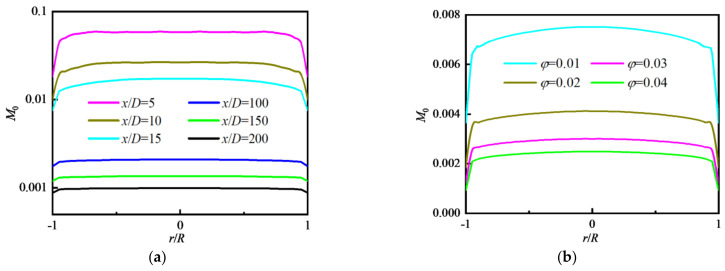
Distribution of *M*_0_ along the radial direction with different *φ* (Re = 9194): (**a**) *φ* = 0.04; (**b**) *x/D* = 117.

Figure 8 shows the distributions of *M*_0_ along the radial direction at different *x/D* and *φ*. In Figure 8a, the values of *M*_0_ are the largest in the inlet area of the tube (*x/D* = 5), and gradually decrease along the flow direction due to the particle coagulation. *M*_0_ in the core area basically presents a uniform distribution. In Figure 8b, the values of *M*_0_ decrease with the increase in *φ*, which is the same as the conclusion given in Figure 7.

#### 4.2.2. Distribution of Particle Diameter

**Figure 9:**
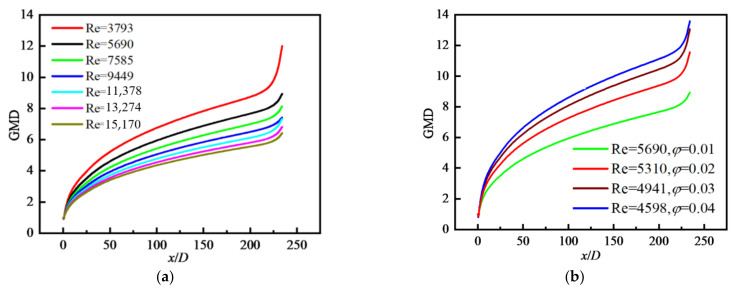
Distribution of GMD along the flow direction with different *φ*: (**a**) *φ* = 0.01; (**b**) at fixed Re and *φ*.

The geometric mean diameter of particle (GMD) is defined in Equation (21). The particles with same size are initially distributed at the inlet, and the GMD will change because of coagulation when particles flow through the tube. The variation in GMD along the flow direction is shown in Figure 9a where *φ* = 0.01. We can see that GMD increases along the flow direction because of particle coagulation. GMD decreases with increasing Re, for example, the value of GMD for Re = 3793 is twice that of GMD for Re = 15,170 at the outlet. The reason is that the larger the Re is, the shorter the residence time of particles in the tube is, and the smaller the possibility of particle collision and coagulation is, so the smaller the value of GMD is. Figure 9b shows the variation in GMD along the flow direction when Re and *φ* change at the same time. It can be seen that the value of GMD increases with increasing *φ*, but with decreasing Re. In the case of large *φ*, the possibility of particle collision and coagulation is high, resulting in large GMD.

**Figure 10:**
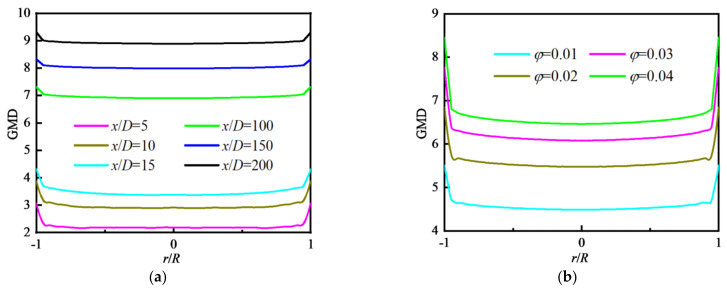
Distribution of GMD along the radial direction with different *φ* (Re = 9194): (**a**) *φ* = 0.04; (**b**) *x/D* = 117.

Figure 10 shows the distributions of GMD along the radial direction at different *x/D* and *φ*. In Figure 10a, the values of GMD are the minimum in the inlet area of the tube (*x/D* = 5), and gradually increase along the flow direction due to the particle coagulation. GMD in the core area basically presents a uniform distribution. In Figure 10b, the values of GMD increase with the increase in *φ*, which is the same as the conclusion given in Figure 9b.

#### 4.2.3. Distribution of Particle Polydispersity

**Figure 11:**
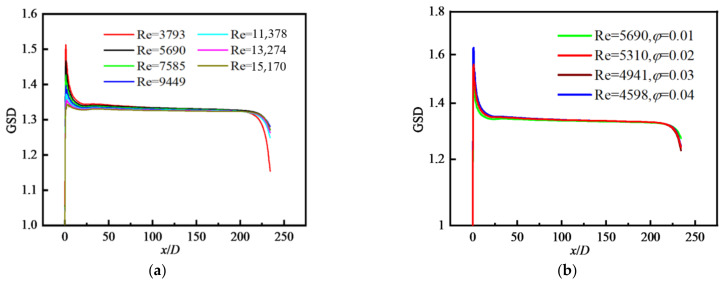
Distribution of GSD along the flow direction with different *φ*: (**a**) *φ* = 0.01; (**b**) at fixed Re and *φ*.

For an initially monodisperse particle field at the inlet, the particle coagulation will result in the change in particle diameter and make the particle field become polydisperse along the tube. The geometric standard deviation of particle diameter (GSD) is defined in Equation (22). The variation in GSD along the flow direction is shown in Figure 11a where *φ* = 0.01. The values of GSD increase sharply at the inlet because the particle coagulation makes initially monodisperse particles change their sizes and particles become polydisperse when particles enter the tube, and then decrease in the inlet area of the tube (*x/D* < 5). Next, the values of GSD remain almost unchanged in the whole tube until at outlet where the values of GSD decrease rapidly again because of the influence of reflux. Figure 11b shows that the effects of Re and *φ* on the variation in GSD along the flow direction are insignificant.

**Figure 12:**
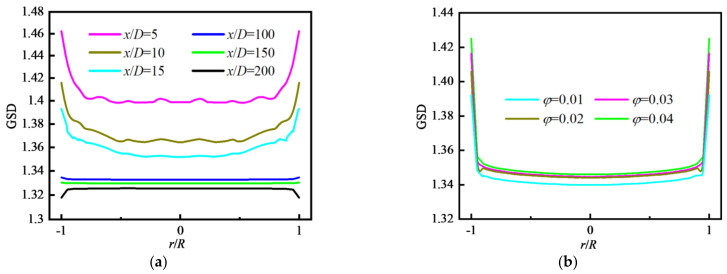
Distribution of GSD along the radial direction with different *φ*: (**a**) *φ* = 0.04; (**b**) *x/D* = 117.

Figure 12a shows the distributions of GSD along the radial direction at different *x/D*. In the inlet area of the tube (*x/D* < 15), the values of GSD decrease along the flow direction, and are unevenly distributed and fluctuate along the radial direction. Downstream (*x/D* > 100), the values of GSD change little along the flow direction and present a uniform distribution along the radial direction. The distributions of GSD along the radial direction for different *φ* are shown in Figure 12b where the *φ* has little effect on GSD except for the case of *φ* = 0.01.

### 4.3. Convective Heat Transfer

**Figure 13:**
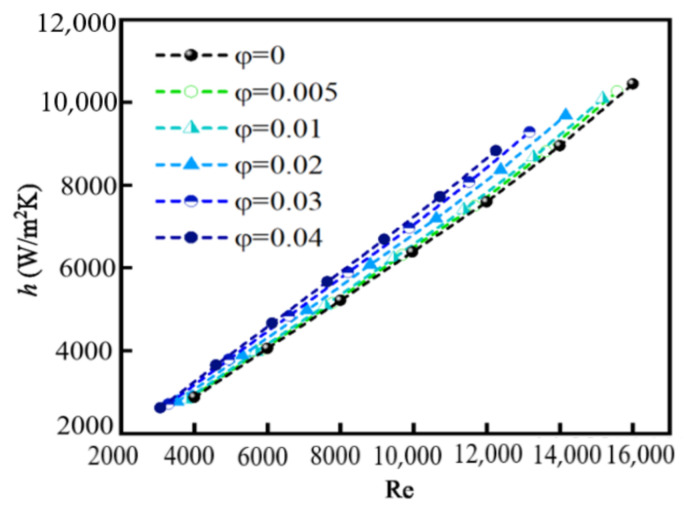
Relationship between *h* and Re for different *φ*.

**Figure 14:**
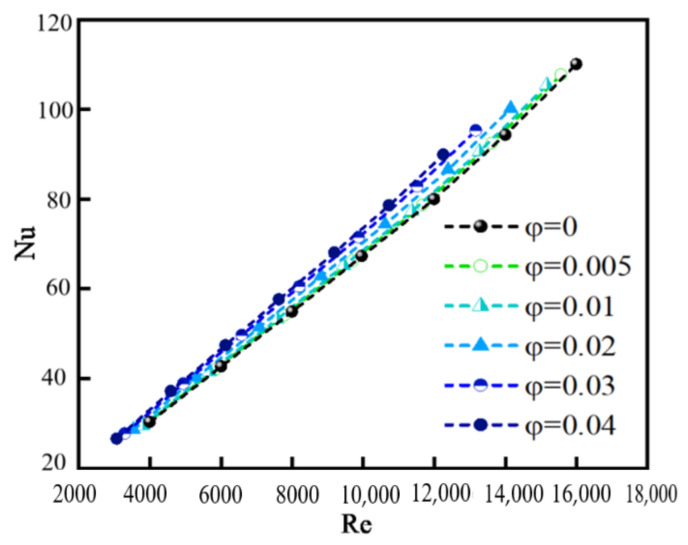
Relationship between Nu and Re for different *φ*.

The convective heat transfer coefficient *h* and Nusselt number Nu are defined in Equation (19). Figure 13 and Figure 14 show the relationship between *h*, *Nu*, and Re for different *φ*. The values of *h* and *Nu* are larger for nanofluids than that for pure water (*φ* = 0), indicating that the addition of nanoparticles in pure water can improve the heat transfer performance, which is consistent with previous conclusions. The reasons for the enhancement of heat transfer performance are attributed to several aspects, for example, the thermal conductivity of the nanofluid is enhanced and hence carries more quantity of heat from the heated surface; mixing effects of particles near the wall; and disordered motion of nanoparticles.

We also can see that the values of *h* and *Nu* increase with the increase in *φ*, which is consistent with the result given by Ali et al. [7]. However, the change in *h* and *Nu* is not large from *φ* = 0.005 to *φ* = 0.04. The reason may be attributed to the fact that the possibility of particle collision and coagulation increases with increasing *φ*, resulting in the increase in particle size. Hemmat and Saedodin [10] indicated that the larger the diameter of the nanoparticle, the smaller the convective heat transfer coefficient. The increase in particle size partially counteracts the effect of heat transfer.

The values of *h* and *Nu* increase nearly linearly when Re changes from 3000 to 16,000. The relationship between *h*, *Nu*, and Re is consistent with previous conclusions [14,15,16]. The fact that the values of *h* and *Nu* are proportional to Re is attributed to the three factors: (1) the disordered movement of particles caused by the turbulent flow is more obvious at high Re, improving the heat transfer; (2) the laminar sublayer near the wall is thinner and the shear stress subsequently increases at high Re; and (3) a greater amount of heat is carried by a faster moving fluid at high Re than a slower moving fluid at low Re.

## 5. Conclusions

The momentum and energy equations of SiO_2_/water nanofluid, flowing through a turbulent tube, together with the population balance equation for nanoparticles were solved numerically with considering the particle convection, diffusion, and coagulation. Some results were validated by comparing with the experimental results. The effect of particle coagulation on the pressure drop ∆*P*, particle distribution, and heat transfer was explored. The main conclusions are summarized as follows:

(1) ∆*P* increases significantly after adding nanoparticles and increases with increasing Re. ∆*P* is proportional to particle volume fraction *φ* because increased viscosity hinders the motion of the nanofluid and more irregular migration of particles. For a specific *φ*, the larger the inlet velocity is, the larger the value of ∆*P* is. When inlet velocity is small, the increase in ∆*P* caused by adding particles is relatively large. The value of ∆*P* increases most obviously compared with the case of pure water when the inlet velocity is 0.589 m/s and *φ* is 0.004.
(2) *M*_0_ decreases along the flow direction. *M*_0_ near the wall is decreased to the original 2% and decreased by about 90% in the central area. For a fixed *φ*, with the increase in Re, *M*_0_ increases and the reduction rate of *M*_0_ along the flow direction decreases. *M*_0_ decreases with increasing *φ* and is the largest in the inlet area, and gradually decreases along the flow direction. *M*_0_ in the core area basically presents a uniform distribution. GMD increases with increasing *φ*, but with decreasing Re because the larger the Re is, the smaller the possibility of particle collision and coagulation is. GMD is the minimum in the inlet area and gradually increases along the flow direction, and basically presents a uniform distribution in the core area. GSD increases sharply at the inlet and decreases in the inlet area, and then remains almost unchanged in the whole tube, finally decreasing rapidly again at the outlet. The effects of Re and *φ* on the variation in GSD along the flow direction are insignificant. In the inlet area, GSD decreases along the flow direction, and is unevenly distributed and fluctuated along the radial direction. Downstream, GSD changes little along the flow direction and presents a uniform distribution along the radial direction. *φ* has little effect on GSD except for the case of *φ* = 0.01.
(3) *h* and *Nu* are larger for nanofluids than that for pure water, and increase with the increase in *φ*. However, the variation in *φ* from 0.005 to 0.04 has little effect on *h* and *Nu* because of particle coagulation. The values of *h* and *Nu* increase nearly linearly when Re changes from 3000 to 16,000 because the disordered movement of particles caused by the turbulent flow is more obvious at high Re, and a greater amount of heat is carried by a faster moving fluid at high Re than a slower moving fluid at low Re.

Future research should further consider the influence of particle breakage after coalescence on pressure drop, particle distribution, and heat transfer of nanofluids.

## Figures and Tables

**Table 1:** *k-ω* SST model constants [24].

Constant	α*_k_*_1_	α*_k_*_2_	α*_ω_*_1_	α*_ω_*_2_	*β*_1_	*β*_2_	*γ*_1_	*γ*_2_	β^*^	*a*_1_	*b*_1_	*c*_1_
Value	0.85	1.0	0.5	0.856	0.075	0.0828	5/9	0.44	0.09	0.31	1.0	10.0

**Table 2:** Thermo-physical properties of the fluid and SiO_2_ nanoparticle at a temperature of 298 K and under normal circumstances.

Thermo-Physical Properties	*ρ* (kg/m^3^)	*μ* (kg/m∙s)	Thermal Conductivity *k* (W/m∙K)	Specific Heat Capacity *c_p_*(J/kg∙K)
Water	997.048	0.00089	0.6072	4182
SiO_2_ (65 nm)	2200	\	1.38	733

**Table 3:** Effective thermo-physical parameters of the nanofluid under normal circumstances.

Volume Fraction *φ*	$\rho_{nf}(\mathrm{kg}/m^{3})$	$C_{p,nf}(J/\mathrm{kg}\cdot K)$	$\mu_{nf}(\mathrm{kg}/m\cdot s)$	Pr	$k_{nf}(W/m\cdot K)$
0 (base fluid)	997.048	4182	0.00089	6.13	0.6027
0.005	1003.06	4144	0.00092	6.25	0.6099
0.01	1009.07	4107	0.00095	6.38	0.6126
0.02	1021.11	4033	0.00103	6.70	0.6181
0.03	1033.14	3962	0.00112	7.09	0.6236
0.04	1047.17	3892	0.00122	7.65	0.6292

**Table 4:** Grid independence test of base fluid at Re = 8000.

	Number of Cells	Node (a × b × c)	∆*P* (Pa)	*h*_mean_(W/m^2^K)
Grid 1	864,000	12 × 9 × 1500	4673.6	5478.9
Grid 2	2,400,000	20 × 15 × 1500	4679.8	5188.42
Grid 3	3,456,000	24 × 18 × 1500	4706.6	5185.44
Grid 4	240,000	20 × 15 × 150	4688.9	5211.87

## Data Availability

Not applicable.

## Abbreviations

*A*	cross-sectional area of the tube
*c_p,nf_*	nanofluid specific heat capacity
*d_f_*	equivalent diameter
*d_p_*	particle diameter
*D*	tube diameter
*D_B_*	Brownian diffusion coefficient
*D_k_*	effective diffusion of k
*D_ω_*	effective diffusion of ω
*E*	total energy
*G*	turbulent kinetic energy generation rate
*h*	convective heat transfer coefficient
*k*	turbulent kinetic energy
*k_B_*	Boltzmann constant
*k_nf_*	nanofluid thermal conductivity
*L*	tube length
*m_k_*	moment
*M*	molecular weight of the base fluid
*n*	particle size distribution function
*N_A_*	Avogadro constant
*R_ij_*	Reynolds stress
**S**	measure of the strain rate tensor
*S_k_*	internal source term of k
*S_ω_*	internal source term of ω
*t*	time
*T*	nanofluid temperature
*u_i_*	nanofluid velocity
*v*	particle volume
*β*	particle coagulation kernel function
*μ*	dynamic viscosity
*μ_nf_*	nanofluid dynamic viscosity
*μ_t_*	turbulent dynamical viscosity
*ρ*	density
*ρ_nf_*	nanofluid density
*ν_t_*	turbulent diffusion coefficient
*φ*	volume fraction of nanoparticles
*ω*	turbulent dissipation rate
